# Chicory Extract Alleviates Anthracycline-Induced Cardiotoxicity by Inhibiting Mitochondrial Damage via the UCP2/NLRP3 Pathway

**DOI:** 10.3390/ijms27031557

**Published:** 2026-02-05

**Authors:** Yifei Rao, Yu Wang, Yadi Liu, Jinjian Huang, Xueli Ding, Zhijian Lin, Bing Zhang, Xiaomeng Zhang

**Affiliations:** 1 School of Chinese Materia Medica, Beijing University of Chinese Medicine, Beijing 102488, China; ryf9696@163.com (Y.R.); wangyuxh@163.com (Y.W.); liuyadi000888@163.com (Y.L.); huangjj@bucm.edu.cn (J.H.); dingxueli202108@163.com (X.D.);; 2 School of Traditional Chinese Medicine, Jiangxi University of Chinese Medicine, Nanchang 330004, China

**Keywords:** chicory, doxorubicin-induced cardiotoxicity, mitochondrial damage, UCP2/NLRP3 pathway

## Abstract

Doxorubicin (Dox)-induced cardiotoxicity (DIC) was characterized by severe myocardial damage that might progress to irreversible heart failure. There were limited options available for the prevention and treatment of DIC. Chicory (*Cichorium intybus* L.) has demonstrated notable cardioprotective effects. However, its potential to mitigate DIC remains unexplored. This study aimed to assess the therapeutic potential of chicory in alleviating DIC and elucidate its active ingredients and potential molecular mechanism. Male Sprague-Dawley (SD) rats were used to construct DIC models. The rats were prophylactically gavaged chicory to evaluate the therapeutic effect of chicory on DIC. The UPLC-QExactivePlus system was used for the subsequent analysis of heart tissue samples to reveal the potential active ingredients of chicory. The binding of chicory components to uncoupling protein 2 (UCP2) and NOD-like receptor thermal protein domain-associated protein 3 (NLRP3) was validated using surface plasmon resonance (SPR). Highly binding ingredients were then utilized in an H9c2 cell model to validate underlying mechanisms. Chicory alleviated Dox-induced cardiac dysfunction and myocardial structural injury, and reversed mitochondrial damage. These protective effects may be attributed to its activation of UCP2 and inhibition of NLRP3 signaling, thereby attenuating Dox-induced cardiac oxidative damage and inflammatory infiltration. Additionally, a total of 15 chemical compositions of chicory into rat heart tissue were characterized. SPR validation demonstrated that nine compounds targeting UCP2 and NLRP3 increased survival rates in Dox-induced H9c2 cells, reduced oxidative and inflammatory levels, and improved mitochondrial function. Chicory could effectively alleviate DIC by reducing oxidative stress, inflammation, and preserving mitochondrial function. These findings offer a novel insight into chicory’s clinical relevance in DIC management. Targeting UCP2 to regulate the NLRP3 pathway highlights chicory as a promising therapeutic strategy for preventing and treating DIC.

## 1. Introduction

Anthracycline chemotherapy has improved the treatment outcomes of various solid and hematological malignancies, extended patient survival and improved quality of life. However, treatment-associated adverse effects, especially cardiotoxicity, have greatly restricted its clinical application [1]. Clinically, Dox was the most commonly used, DIC was characterized by left ventricular dysfunction, ventricular wall thickening, arrhythmias, and heart failure [2]. Currently, there are limited options available for preventing or treating anthracycline-induced cardiotoxicity. Dexrazoxane is the only agent the Food & Drug Administration approved to prevent anthracycline-induced cardiotoxicity [3]. Therefore, there was a pressing demand for the identification and development of safe, effective strategies to mitigate DIC.

*Cichorium intybus* L., commonly known as chicory, is a perennial herb of the Asteraceae family. It holds a significant place in traditional Chinese medicine (TCM) and is also utilized in Uighur ethnomedicine [4]. Various parts of the plant, including its roots, leaves, and seeds, are used medicinally. According to the Chinese Pharmacopoeia, chicory is reputed to purify the liver and gallbladder, strengthen the stomach, and exert diuretic and anti-inflammatory effects [5]. Modern pharmacological studies have identified a rich profile of phytocompounds in chicory, such as inulin, sesquiterpene lactones, Caffeic acid derivatives, fats, proteins, hydroxycoumarins, flavonoids, alkaloids, sterols, and terpenoids. These constituents contribute to its diverse biological activities, including hepatoprotective, anti-inflammatory, antioxidant, sedative, immunomodulatory, hypolipidemic, antidiabetic, and cardiovascular protective effects [6]. Recent studies have further reported that chicory offers protective benefits against various forms of cardiac injury, such as drug-induced cardiotoxicity, myocardial ischemia–reperfusion injury, and diabetic cardiomyopathy [7,8,9]. These findings support its potential as a therapeutic agent for DIC. Research suggests that its cardioprotective effects may be mediated through the inhibition of oxidative stress and pro-inflammatory cytokines [10]. However, the precise pharmacological mechanisms of chicory against DIC have not been fully elucidated.

Mitochondria serve as the specific target organelle for DIC, and mitochondrial dysfunction has recently been recognized as a pivotal element in the development of DIC [11]. Current research indicates that mitochondrial dysfunction triggered cyclic reactive oxygen species (ROS) release, activated the NLRP3 inflammasome and induced inflammatory damage to cardiac tissue, which was the core mechanism underlying DIC [12]. UCP2, as a mitochondrial inner membrane protein, could catalyze the translocation of protons across the inner mitochondrial membrane and decrease ROS production [13]. DOX directly downregulated UCP2 expression in the heart, resulting in mitochondrial dysfunction and oxidative damage to cardiomyocytes [14]. However, UCP2 overexpression could exert cardioprotection by attenuating ROS generation [15]. Additionally, UCP2-mediated mitochondrial ROS release activates the NLRP3 inflammasome, and releases various pro-inflammatory factors, including interleukin-1*β* (IL-1*β*) and interleukin-18 (IL-18), resulting in inflammatory myocardial injury [16]. Concurrently, elevated levels of inflammatory factors exacerbated the state of oxidative stress. This interaction formed a vicious, self-amplifying cycle between oxidative stress and the inflammatory cascade. Ultimately, this cycle triggered cardiomyocyte apoptosis, thereby propagating and intensifying cardiac damage [17]. Those findings strongly suggested that targeting the UCP2/NLRP3 signaling pathway might represent a promising therapeutic strategy to alleviate the progression and severity of DIC. This study investigates the cardioprotective effects of chicory by examining its regulation of the UCP2/NLRP3 pathway. Mass spectrometry (UPLC-QExactivePlus) and SPR technology are used to analyze the active constituents of chicory, and rat models as well as rat cardiomyocyte H9c2 are employed to verify the action mechanisms of chicory.

## 2. Results

### 2.1. Chicory Ameliorated DOX-Induced Cardiac Dysfunction and Injury in Rats

**Figure 1:**
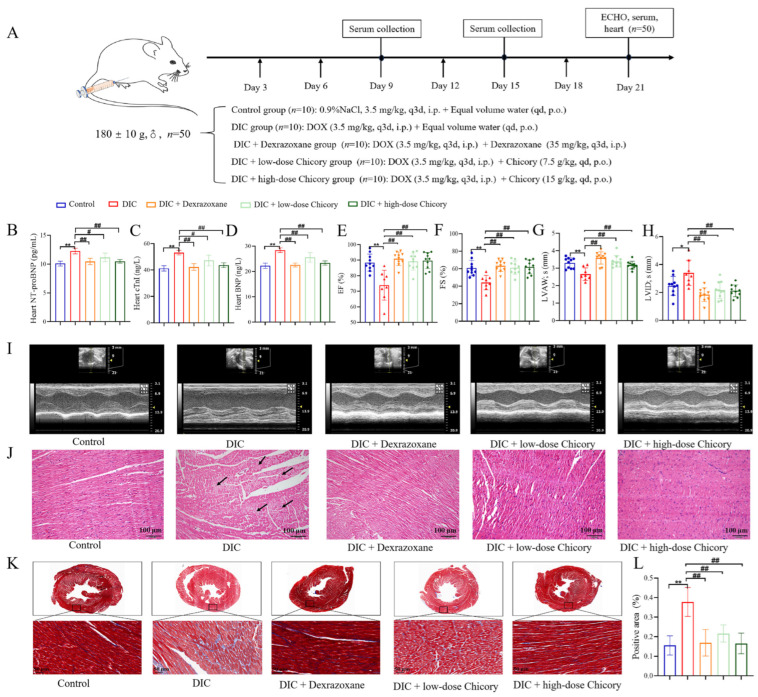
Chicory could effectively mitigate DIC. (**A**) Flowchart of the experiment. (**B**–**D**) Quantitative analysis of NT-proBNP, cTnI and BNP in heart tissue. (**E**–**H**) Quantitative analysis of EF, FS, LVAW; s and LVID; s. (**I**) Representative images of echocardiographic measurements in each group. (**J**) Representative HE staining images in each group were captured at ×200 magnification. (**K**,**L**) The percentage of collagen fibers quantified Masson staining from heart sections of each group and the positive area. Images were captured at ×400 magnification. Compared with the control group, * *p* < 0.05, ** *p* < 0.01. Compared with the DIC group, ^#^ *p* < 0.05, ^##^ *p* < 0.01.

We treated rats with two distinct doses of chicory to evaluate its therapeutic effect on cardiac dysfunction in DIC rats. Drug treatment was administered as indicated in the schematic plan (Figure 1A). Cardiac troponin I (cTnI), Brain natriuretic peptide (BNP), and N-terminal pro-B-type natriuretic peptide (NT-proBNP) were used to assess the status of cardiac injury. From the results of Figure 1B–D, the levels of cTnI, BNP, and NT-proBNP in heart tissue were significantly reduced after chicory treatment. Echocardiography (ECHO) was applied to assess the degree of cardiac injury from the aspect of cardiac function. As shown in Figure 1E–I, ECHO showed that the cardiac function of the DIC group was impaired, whereas an improvement in cardiac function was observed in DIC rats treated with chicory, characterized by increases in ejection fraction (EF), fractional shortening (FS) and left ventricular anterior wall thickness in systole (LVAW; s), and decreases in left ventricular internal diameter in systole (LVID; s). Furthermore, we applied H&E staining and Masson staining to evaluate the structural changes in the heart tissue in each group of rats. As shown in Figure 1J, the myocardial structure in the control group was clear, the myocardial cell tissue was orderly, and no inflammatory cell infiltration was observed. In the DIC group, the myocardial arrangement was irregular, myocardial fibers were broken, and inflammatory cell infiltration was prominent. After intervention with chicory, the myocardial structure and inflammatory infiltration in the DIC rats were significantly improved. Masson staining quantitative analysis was performed to evaluate the deposition of collagen fibers in the heart. As shown in Figure 1K,L, compared with the control group, the collagen fibers in the interstitial tissue of the DIC group were enhanced considerably and distributed in a disordered manner. Compared with the DIC group, the collagen fibers in the interstitial tissue of the DIC + low-dose chicory group and DIC + high-dose chicory group were less. These results collectively demonstrated that chicory administration led to a marked attenuation of both functional and structural cardiac damage induced by DOX in rats.

### 2.2. Chicory Restores Mitochondrial Function Through UCP2/ROS/NLRP3 Pathway to Attenuate Dox-Induced Oxidative Inflammatory Damage

**Figure 2:**
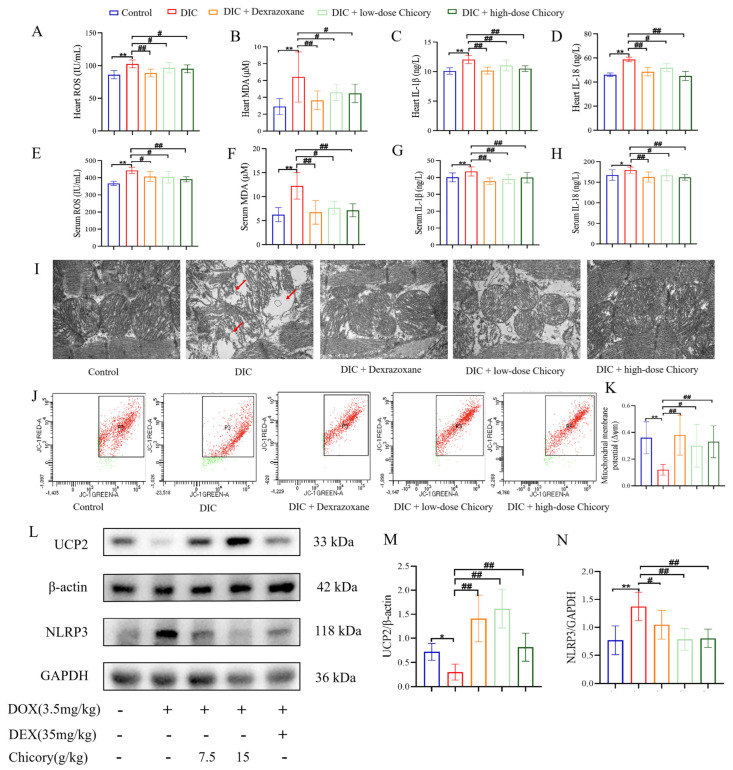
Chicory inhibits DOX-induced ROS production, mitochondrial damage, and UCP2/NLRP3 pathway activation. (**A**–**D**) Quantitative analysis of ROS, MDA, IL-1*β* and IL-18 levels in heart tissue. (**E**–**H**) Quantitative analysis of ROS, MDA, IL-1*β* and IL-18 levels in serum. (**I**) Representative images of TEM in each group were captured at ×12,000 magnification. (**J**,**K**) Quantitative analysis of MMP level from cardiac single-cell suspension in each group, which used the JC-10 fluorescent probe. (**L**–**N**) WB of the UCP2 and NLRP3 protein expressions in heart tissue and their quantification (*n* = 6). Compared with the control group, * *p* < 0.05, ** *p* < 0.01. Compared with the DIC group, ^#^ *p* < 0.05, ^##^ *p* < 0.01. The area pointed to by the arrow was the clearly visible lesion site.

Research indicates that oxidative stress and inflammatory damage are significant contributors to DIC. We investigated the effects of chicory on cardiac oxidative stress and inflammation levels in rats, by detecting ROS, MDA, IL-1*β* and IL-18 levels. Our findings demonstrated that chicory treatment significantly suppressed the elevated levels of ROS, MDA, IL-1*β*, and IL-18 induced by Dox in both cardiac tissue and serum (Figure 2A–H). Furthermore, mitochondria were identified as the principal sites of ROS generation, and their integrity was crucial. We proceeded to assess the impact of chicory on DOX-induced mitochondrial damage. Transmission electron microscopy (TEM) and mitochondrial membrane potential (MMP) were performed for assessing the mitochondrial structure and function. In DIC rats, cardiomyocytes exhibited pronounced mitochondrial damage, including fragmentation of cristae, focal vacuolization, and rupture of the outer membrane. In contrast, treatment with chicory preserved the normal mitochondrial architecture and enhanced its structural integrity, characterized by a reduction in vacuolation and clarity of cristae (Figure 2I). Then, using the JC-10 fluorescent probe to assess the MMP, we observed that there was a marked decrease in cardiomyocytes in the DIC group, whereas chicory effectively restored the MMP (Figure 2J,K).

UCP2, as a mitochondrial anion carrier protein, could protect against mitochondrial oxidative damage by reducing the production of ROS [18,19]. Research demonstrated that DOX directly induced UCP2 downregulation and led to excessive accumulation of ROS [14]. The overproduction of ROS triggered the activation of the NLRP3 inflammasome, resulting in cardiomyocyte apoptosis and myocardial inflammatory damage [20]. In this study, we examined the expression of UCP2 and NLRP3 in cardiac tissue. The results of the WB experiment showed that DOX significantly reduced the expression of UCP2 and increased the expression of NLRP3, whereas treatment with chicory markedly reversed these changes (Figure 2L,M). These findings suggested that chicory restores mitochondrial function and the UCP2/ROS/NLRP3 pathway, thereby attenuating Dox-induced oxidative inflammatory damage.

### 2.3. Analysis of the Cardioactive Components in Chicory

**Figure 3:**
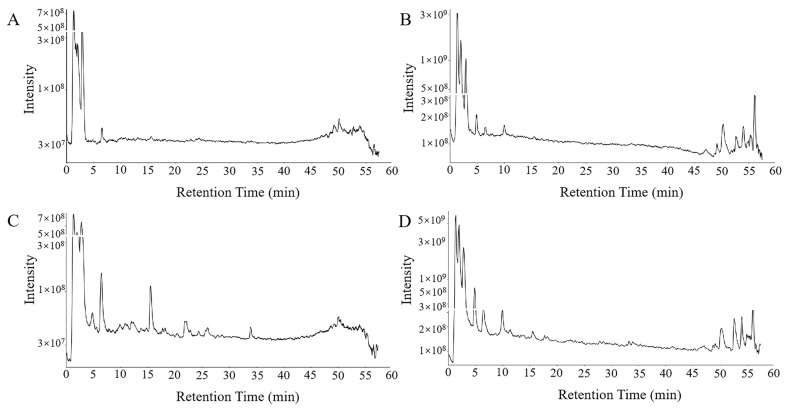
The representative LC-MS chromatograms of the cardioactive components in chicory. (**A**) Base ion chromatogram of blank rat heart tissue homogenate detected in negative mode; (**B**) base ion chromatogram of chicory heart tissue homogenate detected in negative mode; (**C**) base ion chromatogram of blank rat heart tissue homogenate detected in positive mode; (**D**) base ion chromatogram of chicory heart tissue homogenate detected in positive mode.

To investigate the pharmacodynamic foundation and molecular mechanisms of chicory in alleviating DIC, we performed a comprehensive analysis of its cardioactive components through UPLC-QExactivePlus analysis. The total ion chromatograms obtained in both positive and negative ionization modes revealed the presence of chicory’s cardioactive components (Figure 3). By leveraging data from composite mass spectra, consulting comprehensive fragment ion libraries, comparing findings with the published literature, and confirming the results with reference standards, 15 components that entered the heart were identified in chicory; the detailed information is listed in Table 1. The detected compounds included five sesquiterpenes (11*β*,13-Dihydrolactucin, Jacquilenin, Atractylenolide I, Mecheliolide and Parthenolide), two flavonoids (Quercetin-hexoside and Kaempferol-hexoside), three phenylpropanoids (Esculetin, Scopoletin and 7-Methoxycoumarin) and five organic acids (Protocatechuic acid, Caffeic acid, Sinapic acid, p-Hydroxy-cinnamic acid and Azelaic acid), which were confirmed by a comparison with the reference standards (Supplementary Figures S2–S9). These compounds were proposed to serve as the principal bioactive agents, collectively contributing to the mechanistic basis of chicory’s therapeutic action against DIC.

### 2.4. SPR Verified Chicory Targets

**Figure 4:**
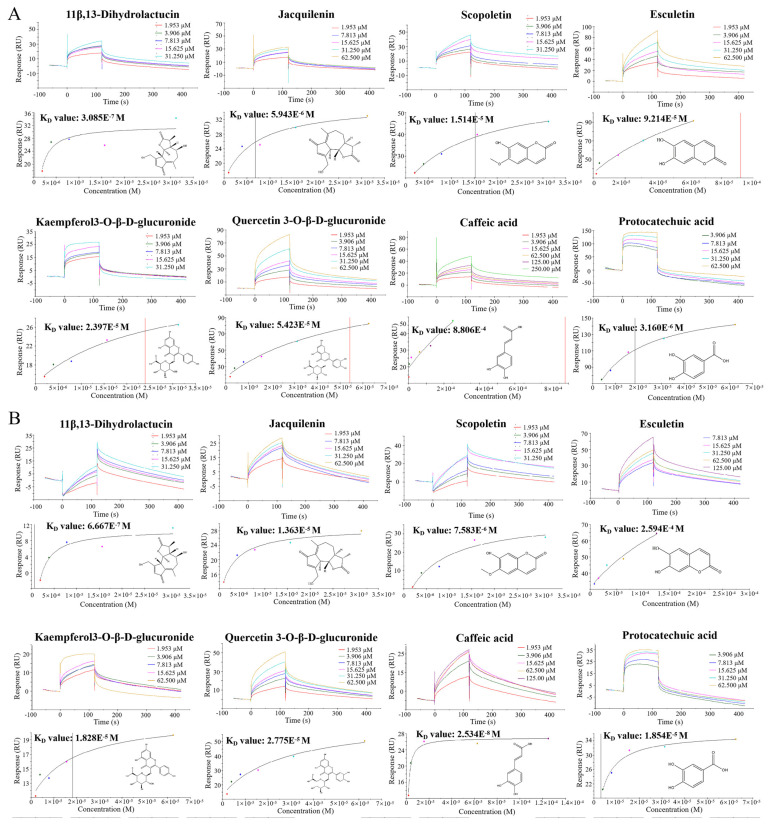
The SPR result of chicory’s active component with UCP2/NLRP3. (**A**) The SPR result of UCP2 and chicory’s active component. (**B**) The SPR result of NLRP3 and chicory’s active component.

The above analyses suggested that the UCP2 and NLRP3 signaling pathways were possible central targets for chicory in mitigating DIC. To elucidate the specific interactions between bioactive constituents and UCP2 or NLRP3 targets, SPR assays were conducted to investigate putative binding and to determine their affinity profiles. The cardioactive components of chicory were selected for analysis with UCP2 and NLRP3 respectively. After completing the solvent correction (Supplementary Table S1 and Figure S12), the 15 small molecules (11*β*,13-Dihydrolactucin, Jacquilenin, Atractylenolide I, Mecheliolide, Parthenolide, Quercetin-hexoside, Kaempferol-hexoside, Esculetin, Scopoletin, 7-Methoxycoumarin, Protocatechuic acid, Caffeic acid, Sinapic acid, p-Hydroxy-cinnamic acid and Azelaic acid) with the different gradient concentrations were tested with UCP2 and NLRP3 in the chip respectively, while the binding energy and the KD value were measured and fitted. Among them, eight molecules exhibited both fast-binding and fast-dissociation modes with UCP2 and NLRP3, and presented strong interactions with UCP2/NLRP3, as shown in Figure 4. Their KD values of UCP2 were ranked as follows: 11*β*,13-Dihydrolactucin (KD = 3.085 × 10^−7^) < Jacquilenin (KD = 5.943 × 10^−6^) < Esculetin (KD = 9.214 × 10^−5^) < Quercetin-hexoside (KD = 5.423 × 10^−5^) < Kaempferol-hexoside (KD = 2.397 × 10^−5^) < Protocatechuic acid (KD = 1.854 × 10^−5^) < Scopoletin (KD = 1.514 × 10^−5^) < Caffeic acid (KD = 8.806 × 10^−4^). Their KD values of NLRP3 were ranked as follows: Caffeic acid (KD = 2.534 × 10^−8^) < 11*β*,13-Dihydrolactucin (KD = 6.667 × 10^−7^) < Scopoletin (KD = 7.583 × 10^−6^) < Protocatechuic acid (KD = 3.160 × 10^−6^) < Quercetin-hexoside (KD = 2.775 × 10^−5^) < Kaempferol-hexoside (KD = 1.828 × 10^−5^) < Jacquilenin (KD = 1.363 × 10^−5^) < Esculetin (KD = 2.594 × 10^−4^). The smaller value of KD meant a stronger affinity and confirmed that the compounds demonstrated specific binding to both the UCP2 and NLRP3 target proteins.

The above analyses suggested that the UCP2 and NLRP3 signaling pathways were possible central targets for chicory in mitigating DIC. To elucidate the specific interactions between bioactive constituents and UCP2 or NLRP3 targets, SPR assays were conducted to investigate putative binding and to determine their affinity profiles. The cardioactive components of chicory were selected for analysis with UCP2 and NLRP3 respectively. After completing the solvent correction (Supplementary Table S1 and Figure S12), the 15 small molecules (11*β*,13-Dihydrolactucin, Jacquilenin, Atractylenolide I, Mecheliolide, Parthenolide, Quercetin-hexoside, Kaempferol-hexoside, Esculetin, Scopoletin, 7-Methoxycoumarin, Protocatechuic acid, Caffeic acid, Sinapic acid, p-Hydroxy-cinnamic acid and Azelaic acid) with the different gradient concentrations were tested with UCP2 and NLRP3 in the chip respectively, while the binding energy and the KD value were measured and fitted. Among them, eight molecules exhibited both fast-binding and fast-dissociation modes with UCP2 and NLRP3, and presented strong interactions with UCP2/NLRP3, as shown in Figure 4. Their KD values of UCP2 were ranked as follows: 11*β*,13-Dihydrolactucin (KD = 3.085 × 10^−7^) < Jacquilenin (KD = 5.943 × 10^−6^) < Esculetin (KD = 9.214 × 10^−5^) < Quercetin-hexoside (KD = 5.423 × 10^−5^) < Kaempferol-hexoside (KD = 2.397 × 10^−5^) < Protocatechuic acid (KD = 1.854 × 10^−5^) < Scopoletin (KD = 1.514 × 10^−5^) < Caffeic acid (KD = 8.806 × 10^−4^). Their KD values of NLRP3 were ranked as follows: Caffeic acid (KD = 2.534 × 10^−8^) < 11*β*,13-Dihydrolactucin (KD = 6.667 × 10^−7^) < Scopoletin (KD = 7.583 × 10^−6^) < Protocatechuic acid (KD = 3.160 × 10^−6^) < Quercetin-hexoside (KD = 2.775 × 10^−5^) < Kaempferol-hexoside (KD = 1.828 × 10^−5^) < Jacquilenin (KD = 1.363 × 10^−5^) < Esculetin (KD = 2.594 × 10^−4^). The smaller value of KD meant a stronger affinity and confirmed that the compounds demonstrated specific binding to both the UCP2 and NLRP3 target proteins.

### 2.5. Chicory Inhibited Inflammation Levels and Mitochondrial Dysfunction in H9c2 Cell

**Figure 5:**
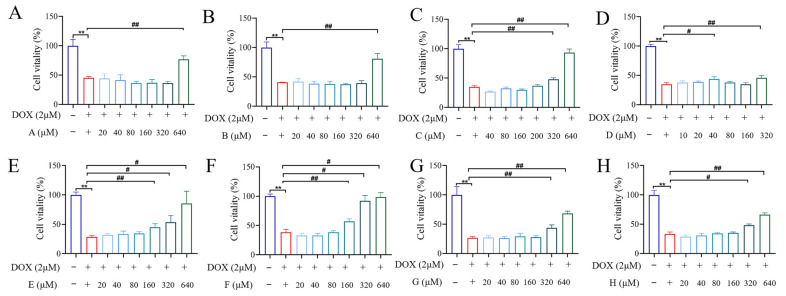
Chicory’s active component could effectively increase the survival rates of DOX-inducing cardiomyocyte (H9c2 cells). (**A**) 11*β*,13-Dihydrolactucin; (**B**) Jacquilenin; (**C**) Scopoletin; (**D**) Esculetin; (**E**) Kaempferol-hexoside; (**F**) Quercetin-hexoside; (**G**) Caffeic acid; (**H**) Protocatechuic acid). Compared with the control group, ** *p* < 0.01. Compared with the DOX group, ^#^ *p* < 0.05, ^##^ *p* < 0.01.

**Figure 6:**
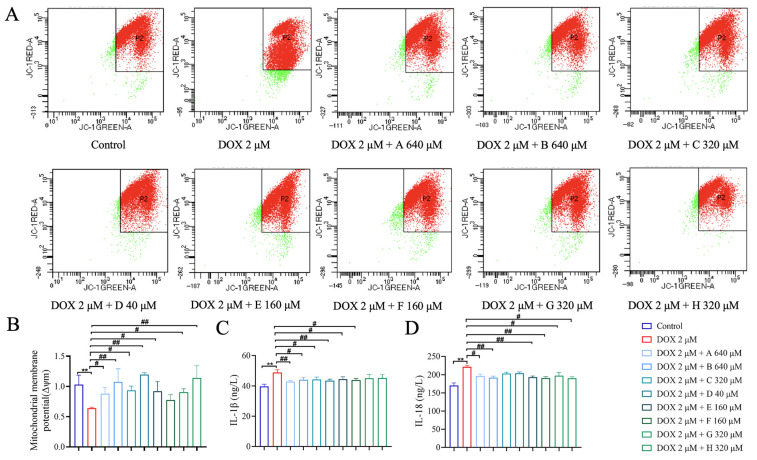
Chicory’s active component could effectively alleviate mitochondrial dysfunction and reduce inflammatory levels in DOX-induced H9c2 cells. (**A**,**B**) Quantitative analysis of MMP level on each H9c2 cell treatment groups, which used the JC-10 fluorescent probe. (A. 11*β*,13-Dihydrolactucin; B. Jacquilenin; C. Scopoletin; D. Esculetin; E. Kaempferol-hexoside; F. Quercetin-hexoside; G. Caffeic acid; H. Protocatechuic acid.) (**C**,**D**) Quantitative analysis of IL-1*β* and IL-18 level in cell supernatant in each group. Compared with the control group, ** *p* < 0.01. Compared with the DOX group, ^#^ *p* < 0.05, ^##^ *p* < 0.01.

To determine appropriate concentrations for DOX and each small molecule, cardiomyocyte survival rates were evaluated. DOX reduced cell survival in a dose-dependent manner (0–40 μM), with an IC_50_ of approximately 1.957 μM. Therefore, 2 μM DOX was selected for inducing cardiomyocyte (H9c2 cells) damage (Supplementary Figure S1). As shown in Figure 5A–H, 11*β*,13-Dihydrolactucin (640 μM) significantly increased the survival rates of DOX-inducing cardiomyocyte (H9c2 cells) damage. Consequently, 640 μM was selected as the treatment concentration of 11*β*,13-Dihydrolactucin. Similarly, the therapeutic concentrations of other small molecules were as follows: Jacquilenin (640 μM), Scopoletin (320 μM), Esculetin (40 μM), Kaempferol-hexoside (160 μM), Quercetin-hexoside (160 μM), Caffeic acid (320 μM) and Protocatechuic acid (320 μM). Those compounds of chicory partially improved the survival rate of DOX-treated H9c2 cells (Figure 5A–H) and significantly reduced IL-1*β* and IL-18 levels (Figure 6C,D). Furthermore, the results from the MMP assessment revealed that cells treated with DOX exhibited a significantly reduced MMP compared to the control group. However, each compound significantly rescued this loss of MMP across the therapeutic concentrations (Figure 6A,B). These findings indicated that chicory alleviated inflammation and mitochondrial dysfunction, underscoring its therapeutic potential against DIC.

## 3. Discussion

This study investigates the protective effects of chicory against DIC in rat and cardiomyocyte models. Chicory exhibited antioxidant and anti-inflammatory properties, as well as exerted ameliorative effects on mitochondrial damage, thereby alleviating DIC. Additionally, chicory’s core components improved cardiomyocyte viability, reduced inflammation and enhanced mitochondrial membrane potential. SPR and in vitro studies indicated that the core chicory component enhances mitochondrial function through the targeting of the UCP2/NLRP3 pathway, thus protecting cardiac function.

Although mechanistic details were still being unraveled, DOX was recognized to function fundamentally as a mitochondrial toxin, and the mitochondrial injury it precipitated was understood to be a central mechanism underlying its cardiotoxicity in DIC [21]. DOX specifically accumulated in mitochondria at concentrations 100-fold higher than in plasma, and mitochondria comprised approximately 30% of the cardiomyocyte volume. Thus, DOX-induced myocardial dysfunction was closely linked with mitochondrial bioenergetics [22]. Mitochondria supported cardiac function by providing essential energy, primarily generating ATP through oxidative phosphorylation and glycolysis to maintain cardiomyocyte viability and contractile activity [23]. Concurrently, they constituted the principal intracellular source of ROS. Mitochondrial impairment disrupted this balance, causing a cascade of excessive ROS production that ultimately triggered oxidative stress [24]. Oxidative stress triggered inflammatory responses, which in turn exacerbated cardiomyocyte injury. This reciprocal aggravation between oxidative stress and inflammation further worsened mitochondrial dysfunction, thereby establishing a self-perpetuating pathological cycle that severely compromised cardiac function [25]. Consequently, the timely reversal of mitochondrial damage and the preservation of mitochondrial integrity were critical for facilitating cardiac recovery.

Chicory treatment significantly attenuated myocardial dysfunction in the DIC rat model. This therapeutic benefit was demonstrated through the suppression of key cardiac injury biomarkers (NT-proBNP, cTnI, and BNP), a reduction in oxidative stress (ROS, MDA) and inflammatory markers (IL-1*β*, IL-18), the amelioration of myocardial pathological damage, and an enhancement in mitochondrial function as reflected by increased membrane potential. However, the well-known feature of TCM is the complex ingredients. Thus, we analyzed the chemical profile of chicory using UPLC-QExactivePlus technology [26]. The main constituents were sesquiterpenes, flavonoids, phenylpropanoids and organic acids. The quantification of the ingredient of chicory is beneficial as a quality control for further drug development. Sesquiterpenes were a major class of secondary metabolites reported in chicory, exhibiting pro-apoptotic activity, anti-inflammatory, antibacterial, antioxidant, hypolipidemic, and hypoglycemic effects [27]. In this study, five sesquiterpenoids were identified from the migrating components of chicory into the heart: 11*β*,13-Dihydrolactucin, Jacquilenin, Atractylenolide I, Mecheliolide and Parthenolide. 11*β*,13-dihydrolactucin has been reported to exhibit the highest anti-inflammatory potential among sesquiterpene lactones. It inhibits Crz1 (calcineurin-responsive zinc finger transcription factor) activation by 54% and modulates the nuclear factor of activated T-cells (NFAT) pathway, thereby regulating inflammatory responses [28]. Two flavonoids, Quercetin-hexoside and Kaempferol-hexoside, were identified. Flavonoids have demonstrated advantages in preventing DIC without interfering with their antitumor efficacy [29]. For instance, Quercetin alleviates cardiotoxicity by inhibiting ROS accumulation and activating the ERK1/2 pathway, while also enhancing chemotherapeutic efficacy [30]. Kaempferol-hexoside has been shown to significantly inhibit multiple pro-inflammatory mediators, including IL-1*β*, NO, PGE2, and LTB4, while upregulating the secretion of the anti-inflammatory cytokine IL-10 [31,32]. Three phenylpropanoids were identified: Esculetin, Scopoletin and 7-Methoxycoumarin. Esculetin has been reported to ameliorate DIC by modulating the expression of Bmi-1 [33]. Scopoletin mediates cardioprotection in isoproterenol-induced myocardial infarction via its inherent antioxidant and anti-inflammatory activities [34]. Five organic acids were identified: Protocatechuic acid, Caffeic acid, Sinapic acid, p-Hydroxy-cinnamic acid and Azelaic acid. Among them, Protocatechuic acid protects against DIC by blocking oxidative stress, attenuating inflammation, and suppressing apoptosis [35]. Caffeic acid counters iron-induced cardiotoxicity by inhibiting angiotensin-converting enzyme activity and modulating the lipid profile [36]. Those provide essential theoretical and experimental support for the treatment of chicory with DIC.

Currently, SPR technology is widely employed due to its ability to accurately characterize the molecular interactions [37]. Notably, SPR suggested that the core components responsible for the beneficial effects of chicory on DIC are 11*β*,13-Dihydrolactucin, Jacquilenin, Scopoletin, Esculetin, Kaempferol-hexoside, Quercetin-hexoside, Caffeic acid and Protocatechuic acid, and suggested that the UCP2/NLRP3 pathway serves as a target for chicory regulation during DIC progression.

UCP2, a key mitochondrial antioxidant protein, was considered a key target for inhibiting oxidative damage in the pathological processes of various cardiovascular diseases [38]. Targeted UCP2 therapy alleviated ROS accumulation and reversed DOX-induced perivascular fibrosis and cardiotoxicity [39], and attenuated free radical damage to help mitochondria antagonize the DIC [40]. Inhibited UCP2 could promote DOX-induced oxidative stress and cardiomyocyte apoptosis [41]. Overexpression of ROS directly triggered NLRP3.

NLRP3 functioned as a cytoplasmic pattern recognition receptor capable of detecting diverse danger-associated molecular patterns and environmental stressors. Its activation within cardiac tissue initiated the assembly of the inflammasome complex. This, in turn, facilitated the proteolytic activation of caspase-1, which catalyzed the maturation and secretion of pro-inflammatory cytokines, notably IL-1*β* and IL-18, ultimately culminating in a potent inflammatory response [42]. Numerous studies have established that DIC was fundamentally an inflammatory condition, pathologically defined by the excessive production of pro-inflammatory cytokines. Consequently, therapeutic strategies aimed at modulating these specific cardiac inflammatory responses emerged as a highly promising direction for managing DIC. Previous studies had reported that chicory possessed significant anti-inflammatory properties, with well-established beneficial effects on various disorders including kidney inflammation [43] and hepatitis [44,45,46]. Nevertheless, the potential function and mechanistic role of chicory in DIC were largely unknown. In the present study, our findings demonstrated that chicory reverses DOX-induced decreases in UCP2 protein expression and decreases the ratios of NLRP3 in cardiac tissue. Furthermore, SPR confirmed that key chicory components directly target UCP2 and NLRP3. Collectively, these results suggest that chicory ameliorates DIC by activating UCP2, inhibiting the NLRP3 pathway, and enhancing mitochondrial function.

## 4. Materials and Methods

### 4.1. Materials and Reagents

Chicory (*Cichorium intybus* L.) was purchased from Xinjiang Minhai Traditional Chinese Medicinal Decoction Pieces Co., Ltd., the batch number 181201 (Xinjiang, China). Dexrazoxane was obtained from Jiangsu Aosaikang Pharmaceutical Co., Ltd. (Nanjing, China). Collagenase II and Dispase II were purchased from Beijing Solarbio Science & Technology Co., Ltd. (Beijing, China). The lipid peroxidation MDA assay kit was obtained from Beyotime Biotech Inc (Shanghai, China). BNP, NT-proBNP, cTnI, ROS, IL-1*β* and IL-18 enzyme-linked immunosorbent assay (ELISA) kits were purchased from Jiangsu Meimian Industrial Co., Ltd. (Yancheng, China). Anti-UCP2, anti-Nlrp3, anti-GAPDH and beta actin polyclonal antibody were all purchased from Proteintech Group (Wuhan, China). 11*β*,13-Dihydrolactucin (B29290), Kaempferol3-O-*β*-D-glucuronide (B50069), Caffeic acid (B20660), Protocatechuic acid (B21614), Parthenolide (B21375), Mecheliolide (B20891), Atractylenolide I (B20054), Sinapic acid (B25310), 7-Methoxycoumarin (B21848), p-Hydroxy-cinnamic acid (B20333) and Azelaic acid (B26383) were purchased from yuanye Bio-Technology (Shanghai, China). Jacquilenin (CFN95434) was obtained from ChemFaces (Wuhan, China). Scopoletin (DST230427-064) was obtained from Chengdu Lemeitian Pharmaceutical Technology Co., Ltd. (Chengdu, China). Esculetin (A0021) was obtained from Chengdu Must Bio-technology Co., Ltd. (Chengdu, China). Quercetin 3-O-*β*-D-glucuronide (RFS-H05611812016) was obtained from Chengdu Ruifen Si Biotechnology Co., Ltd. (Chengdu, China).

### 4.2. Preparation of Chicory Extract

A total of 1.5 kg of chicory was combined with 15 L of distilled water and soaked for 30 min. The mixture was then brought to a boil over high heat, followed by simmering over low heat for 1 h. After filtration through gauze, the first filtrate was collected. Subsequently, 12 L of distilled water was added to the residue, and the decoction process was repeated under the same conditions (boiling followed by 1 h simmering). The second filtrate was obtained through gauze filtration. Finally, the two filtrates were combined and concentrated under reduced pressure to a final volume of 100 mL, yielding a chicory extract with a concentration of 15 g/mL [4,5].

### 4.3. Experimental Animal

Fifty male SD rats (180 ± 10 g) were purchased from Beijing Si Bei Fu Biotechnology (Beijing, China; production license no.: SCXK-2023-0011) and acclimatized for 3 days in the Beijing University of Chinese Medicine animal facility (temperature: 22–26 °C, relative humidity: 50–60%, 12 h light/dark cycle). This study was conducted according to proven guidelines specified by the animal ethics committee of the Beijing University of Chinese Medicine (Beijing, China; Approval NO.: BUCM-2023050504-2147; Approved date: 5 May 2023).

#### 4.3.1. Groups and Treatment

According to the method of a random number table, 50 male SD rats were respectively divided into six groups: (1) control group (*n* = 10); (2) DIC group (*n* = 10); (3) DIC + dexrazoxane group (*n* = 10, 35 mg/kg); (4) DIC + low-dose chicory group (*n* = 10, 7.5 g/kg); (5) DIC + high-dose chicory group (*n* = 10, 15 g/kg). This study used DOX to establish the DIC rat model through intraperitoneal injection at a dose of 3.5 mg/kg every three days. Among them, dexrazoxane was the agent the Food & Drug Administration approved to prevent DIC, and was used as the positive control drug in this study. The dexrazoxane group received intraperitoneal injections of dexrazoxane half an hour before each model induction; chicory aqueous extract was administered by gavage for 6 days prior to the induction of DIC and continued until the end of the experiment. The control group and the DIC group received gavage or injections of saline.

#### 4.3.2. ECHO

ECHO was performed using the Vevo 2100 system (VisualSonics, Toronto, ON, Canada). Rats were anesthetized via intraperitoneal injection of 1.5% sodium pentobarbital. Subsequently, the chest hair was shaved and hair removal gel was applied to minimize ultrasound beam attenuation. Rats were positioned supine on a temperature-controlled platform. M-mode images were acquired at the papillary muscle level. Key cardiac parameters, measured over three consecutive cardiac cycles, included: EF, FS, LVAW; s, LVID; s, left ventricular posterior wall thickness in systole (LVPW; s), and left ventricular volume in systole (LV Vol; s).

#### 4.3.3. Indicator Detection

Blood samples were collected from the abdominal aorta, allowed to stand, and subsequently centrifuged (3500 rpm, 10 min). Snap-frozen myocardial tissue (50 mg) was homogenized in a 400 μL pre-cooled phosphate-buffered saline (PBS) and then centrifuged (5000× *g*, 15 min, 4 °C). Those supernatants were collected to assay the level of cardiac injury biomarkers (cTnI, BNP and NT-proBNP), and oxidative and inflammatory factors (ROS, MDA, IL-1*β* and IL-18) in each group of rats.

#### 4.3.4. Histological Evaluation

Myocardial tissue samples were fixed by immersion in 4% paraformaldehyde and incubated overnight at 4 °C. Subsequently, the fixed tissues were processed through dehydration, embedded in paraffin, and sectioned at a thickness of 3 μm. The obtained sections were stained using standard Hematoxylin & Eosin (H&E) and Masson’ s trichrome protocols for the purpose of identifying histopathological alterations in myocardial structure. Finally, digital images of the stained sections were acquired using a Leica microscope (Leica, Wetzlar, Germany) under 200× and 400× magnification. Following that, regarding the results of Masson staining, the percentage of collagen fiber areas was calculated using Image-Pro Plus 6.0 software.

#### 4.3.5. TEM

Mitochondrial integrity and morphological alterations were assessed by TEM. Briefly, small samples of myocardial tissue were fixed in pre-cooled 2.5% glutaraldehyde, followed by dehydration in a graded ethanol series. Subsequently, samples were embedded, polymerized, and sectioned. Ultrathin sections (60–80 nm thickness) were prepared using a Leica EM UC7 microtome (Leica, Wetzlar, Germany) and stained with 2% uranyl acetate and 2.6% lead citrate, respectively. Finally, images were acquired using a HITACHI HT7800 electron microscope (Dongjing, Japan).

#### 4.3.6. MMP Assay

MMP was determined using the JC-10 MMP detection kit (Beijing Solarbio Science & Technology Co., Ltd., Beijing, China). Firstly, the rat cardiac single-cell suspension was prepared, the fresh heart tissue (100 mg) was digested in digestive solution (a mixture of collagenase II 0.8 mg/mL and neutral protease II 1.25 mg/mL) and shaken for 15 min, repeated 3 times, supernatants were collected and filtered, centrifuged (300× *g*, 10 min), resuspended in JC-10 staining solution and incubated for 30 min at 37˚C in the dark, washed twice and resuspended in 500 μL JC-10 staining buffer, and then measured using a flow cytometer (DMI 3000B, Leica, Wetzlar, Germany). Notably, the gating strategy applied in the experiments was optimized through preliminary studies, in which carbonyl cyanide 3-chlorophenylhydrazone (CCCP) was employed as a depolarizing positive control to ensure precise discrimination of cell populations for MMP analysis. The ratio of red fluorescence intensity (JC-10 aggregates) to green fluorescence intensity (JC-10 monomers) in the cells served as a direct indicator of the MMP levels.

#### 4.3.7. Western Blot (WB) Analysis

Protein extraction was performed from rat myocardial tissue samples, and the total protein concentration was determined. A standardized amount (20 μg) of each protein sample was then loaded and separated by sodium dodecyl sulfate–polyacrylamide gel electrophoresis according to established protocols. Following electrophoresis, the separated proteins were electrophoretically transferred onto polyvinylidene fluoride membranes (Biorigin (Beijing) Inc., China). The membranes underwent a blocking step using ncmblot blocking buffer to prevent nonspecific binding, the blocked membranes were then incubated at 4 °C overnight with primary antibodies against UCP2 (1:2000), Nlrp3 (1:1000), GAPDH (1:50,000), and *β*-actin (1:5000 dilution) were used as the loading control. Following three washes with TBST buffer, the membranes were incubated for 2 h at room temperature with horseradish peroxidase (HRP)-conjugated secondary antibodies at a dilution of 1:5000. After another round of TBST washes, the specific protein bands were visualized by applying enhanced chemiluminescence (ECL) substrate. The resulting luminescent signals were captured and subjected to semi-quantitative densitometric analysis using ImageJ 2.0 software.

### 4.4. Analysis of Heart Tissue Chemical Composition

SD rats (Male, 220 ± 10 g) were administered chicory extract (30 g/kg·bw^−1^) by gavage every 12 h for a total of 6 times. The rats were then anesthetized with a 0.5% sodium pentobarbital solution at 2 h after last administration. Abdominal aorta blood sampling was followed by execution and dissection of heart tissue. Heart tissue homogenate was precipitated by adding triple acetonitrile, after which the acetonitrile was evaporated and redissolved in methanol–water (7:3), and centrifuged (14,000 rpm, 5 min, 4 °C) for analysis.

The analysis of heart tissue chemical composition was achieved by a UPLC-QExactivePlus system with an ACQUITY UPLC HSS T3 column (Waters, 2.1 × 100 mm, 1.8 μm). The mobile phase consisted of 0.1% formic acid in water (A) and methanol (B), delivered at a flow rate of 0.2 mL/min. The injection volume was 2 µL, with the column temperature maintained at 30 °C and the sample chamber temperature set at 10 °C. The gradient elution program was as follows: 0–3 min (90% A); 3–15 min (75% A); 15–29 min (60% A); 29–38 min (55% A); 38–50 min (30% A); 50–55 min (0% A); 55–60 min (0% A).

### 4.5. SPR Analysis

The SPR assay was conducted using a Biacore T200 instrument (Cytiva, Marlborough, MA, USA) equipped with a CM5 sensor chip (29-1049-88, Cytiva, Marlborough, MA, USA). Following chip activation, the recombinant proteins UCP2 (RPC586Hu01, Cloud-Clone Corp., Wuhan, China) and NLRP3 (RPK115Hu01, Cloud-Clone Corp., Wuhan, China) were immobilized onto separate flow cells via amine coupling, as directed by the wizard function in the instrument control software (Supplementary Figures S10 and S11). Each monomeric compound was initially prepared as a 20 mM stock solution in DMSO. This stock was subsequently diluted with a running buffer containing 5% DMSO to yield a 1 mM working solution. Finally, this working solution was subjected to a series of gradient dilutions for the precise determination of the equilibrium dissociation constant (KD). The KD values were calculated using the Biacore T200 3.2 evaluation software, reflecting the binding strength between compounds and target proteins, and compounds with a high binding strength were screened for validation at the cellular level.

### 4.6. Cell Culture and Treatments

H9c2 cells were acquired from the Cell Resource Center of the Institute of Basic Medical Sciences (Beijing, China) and were maintained under standard cell culture conditions. They were cultured in Dulbecco’s Modified Eagle Medium (DMEM) supplemented with 10% fetal bovine serum (FBS) and 1% penicillin–streptomycin (P/S), in a humidified atmosphere with 5% CO_2_ at 37 °C, subcultured every 2–3 days. The cells were assigned as the following groups: the control group, the DOX (2 μM) group, the DOX (2 μM) + each chicory active ingredient groups; the cells in each group were incubated for 24 h, and the cell viability was measured by using the Cell Counting Kit-8 assay (Beijing Solarbio Science & Technology Co., Ltd., Beijing, China). The levels of inflammatory factors (IL-1*β* and IL-18) in the cell supernatant were measured as described above for the ELISA kit assay. MMP was also determined using the JC-10 MMP detection kit.

### 4.7. Statistical Analysis

Data were analyzed with IBM SPASS Statistics 20 (IBM Corp, Armonk, NY, USA). All values are presented as the mean ± standard deviation (SD). For the analysis of experimental data, using one-way ANOVA for data with normal distribution, the nonparametric test was performed for non-normally distributed data. Statistical significance was set at *p* < 0.05 and *p* < 0.01. All figures were produced using GraphPad Prism 9.0.0 (GraphPad Software Inc., San Diego, CA, USA).

## 5. Conclusions

**Figure 7:**
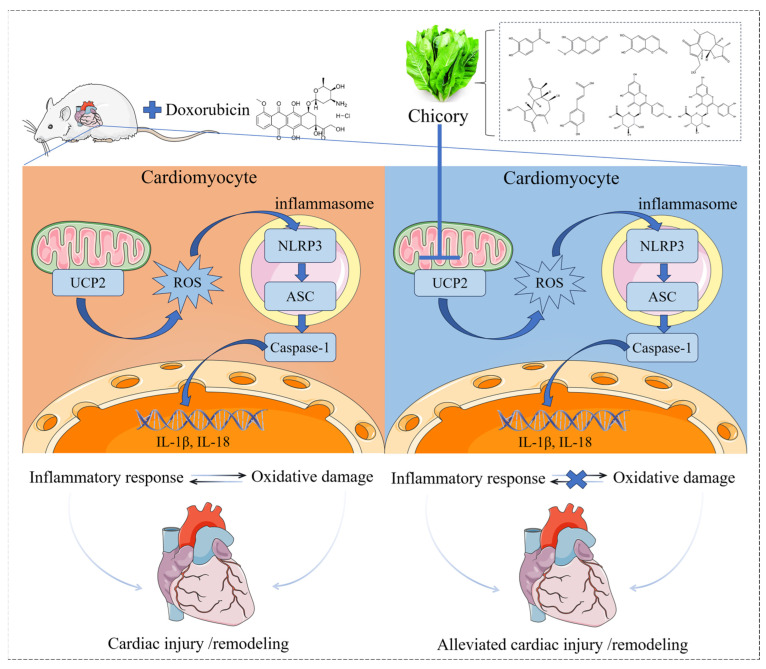
Schematic of a possible molecular mechanism by which chicory improves DIC. Arrows indicated the overall mechanism pathway, and blue X denoted the inhibition of the pathway.

Chicory exerted significant cardioprotective effects against DIC, achieved through the reduction in oxidative stress and inflammation, coupled with the preservation of the mitochondrial structure and function. By demonstrating its regulatory action on the UCP2/NLRP3 pathway, these findings offered a novel mechanistic insight into chicory’s potential clinical relevance for DIC management. This positions the regulation of the UCP2/NLRP3 axis as a key mechanism, highlighting chicory as a promising therapeutic strategy for the prevention and treatment of DIC (Figure 7).

## Figures and Tables

**Table 1:** Identification of compounds of chicory into the heart tissue.

No.	RT (min)	Identification	Molecular Formula	Ion Mode	Calculated (*m*/*z*)	Measured (*m*/*z*)	Error (ppm)	MS^2^
1	7.34	Protocatechuic acid *	C_7_H_6_O_4_	[M − H]^−^	154.02585	153.01857	−4.95	109.02843
2	15.66	Esculetin *	C_9_H_6_O_4_	[M + H]^+^	178.02641	179.03407	−1.11	151.03925, 133.02870, 123.04445
3	16.68	Caffeic acid *	C_9_H_8_O_4_	[M − H]^−^	180.04172	179.03444	−3.02	135.04417, 107.04848
4	18.22	11*β*,13-Dihydrolactucin *	C_15_H_18_O_5_	[M + H]^+^	278.11521	279.12244	0.02	261.11230, 243.10161, 215.10690, 187.07558, 159.08061
5	22.17	p-Hydroxy-cinnamic acid *	C_9_H_8_O_3_	[M − H]^−^	164.04652	163.03925	−3.69	119.04913
6	22.91	Scopoletin *	C_10_H_8_O_4_	[M + H]^+^	192.04228	193.04956	0.13	178.02623, 137.05994, 133.02866
7	25.60	Sinapic acid *	C_11_H_12_O_5_	[M − H]^−^	224.06848	223.0612	0.99	208.03777, 193.01387, 179.07094, 164.04724
8	30.72	Jacquilenin *	C_15_H1_8_O_4_	[M + H]^+^	262.12029	263.12756	−0.85	245.11806, 217.12288, 189.09145, 161.09644, 133.10156, 105.07047
9	31.20	Quercetin-hexoside *	C_21_H_18_O_13_	[M − H]^−^	478.07578	477.06760	0.92	301.03632, 178.99808, 151.00285, 107.01244
10	33.93	Azelaic acid *	C_9_H_16_O_4_	[M − H]^−^	188.10465	187.09713	−1.11	169.08626, 141.86725, 125.09617, 97.06464
11	35.60	Kaempferol-hexoside *	C_21_H_18_O_12_	[M − H]^−^	462.08086	461.07468	2.28	285.04114, 257.04520, 229.05054, 175.02379
12	48.09	Atractylenolide I *	C_15_H_18_O_2_	[M + H]^+^	230.13094	231.13821	1.12	213.12743, 203.14330, 185.13269, 157.10139, 143.08569, 105.07043
13	48.84	7-Methoxycoumarin *	C_10_H_8_O_3_	[M + H]^+^	176.04755	177.05481	−2.75	149.05997, 107.04963
14	53.34	Mecheliolide *	C_15_H_20_O_2_	[M + H]^+^	232.14664	233.15391	1.56	215.14320, 187.14835, 159.11685, 131.08589, 105.07039
15	56.45	Parthenolide *	C_15_H_20_O_3_	[M + H]^+^	248.14154	249.14883	−3.40	193.12283, 179.07042, 161.05994, 133.06500

Note: * Indicates components confirmed by standards.

## Data Availability

The original contributions presented in this study are included in the article/Supplementary Material. Further inquiries can be directed to the corresponding authors.

## Supplementary Materials

The following supporting information can be downloaded at: https://www.mdpi.com/article/10.3390/ijms27031557/s1.
