# Supplementary material for: Chicory Extract Alleviates Anthracycline-Induced Cardiotoxicity by Inhibiting Mitochondrial Damage via the UCP2/NLRP3 Pathway
Source: Int J Mol Sci. 2026 Feb 5;27(3):1557. doi: 10.3390/ijms27031557 (PMC12898394; doi:10.3390/ijms27031557)
Supplement: Supplementary file 1 [file ijms-27-01557-s001.zip › ijms-4070383-supplementary.pdf]

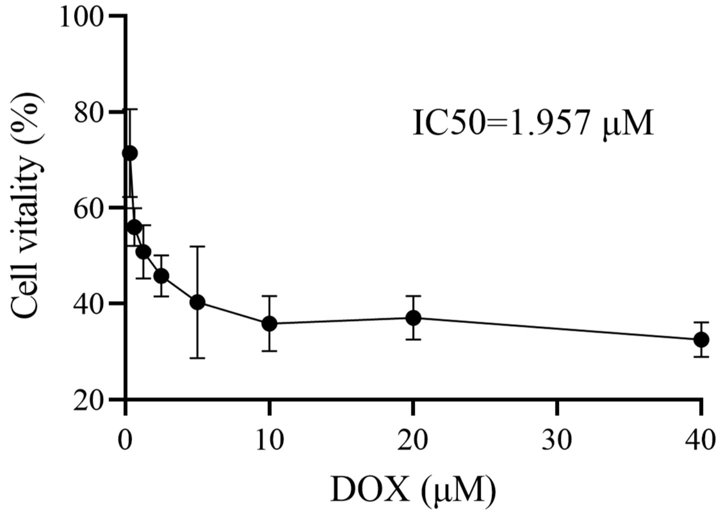

Supplementary Figure S1. Cell viability at various DOX concentrations (*n* = 6).

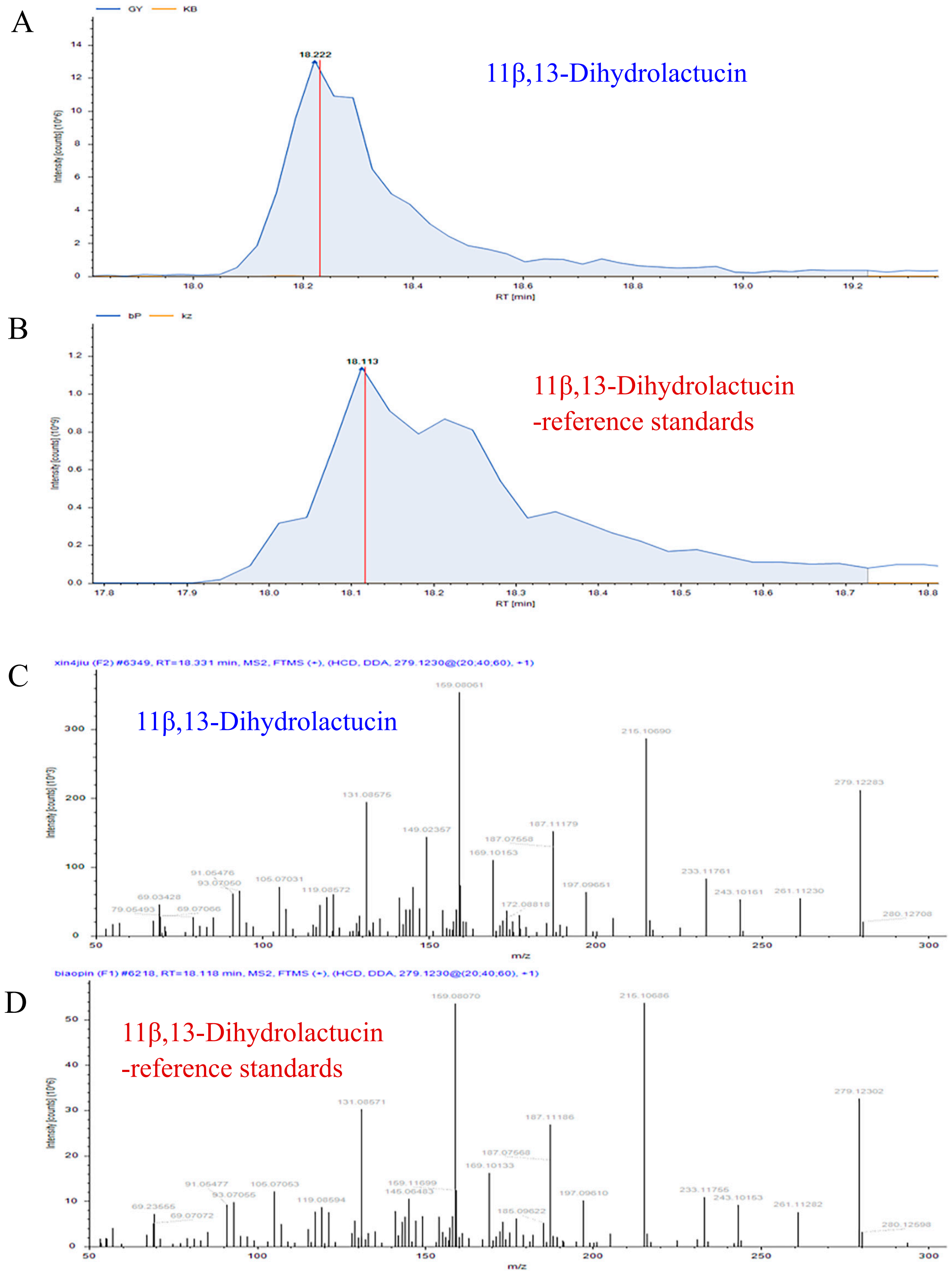

Supplementary Figure S2. (**A**) The ion chromatogram of 11β,13-Dihydrolactucin detected in positive mode; (**B**) The ion chromatogram of 11β,13-Dihydrolactucin-reference standards detected in positive mode; (**C**) The secondary mass spectra of 11β,13-Dihydrolactucin detected in positive mode; (**D**) The secondary mass spectra of 11β,13-Dihydrolactucin-reference standards detected in positive mode.

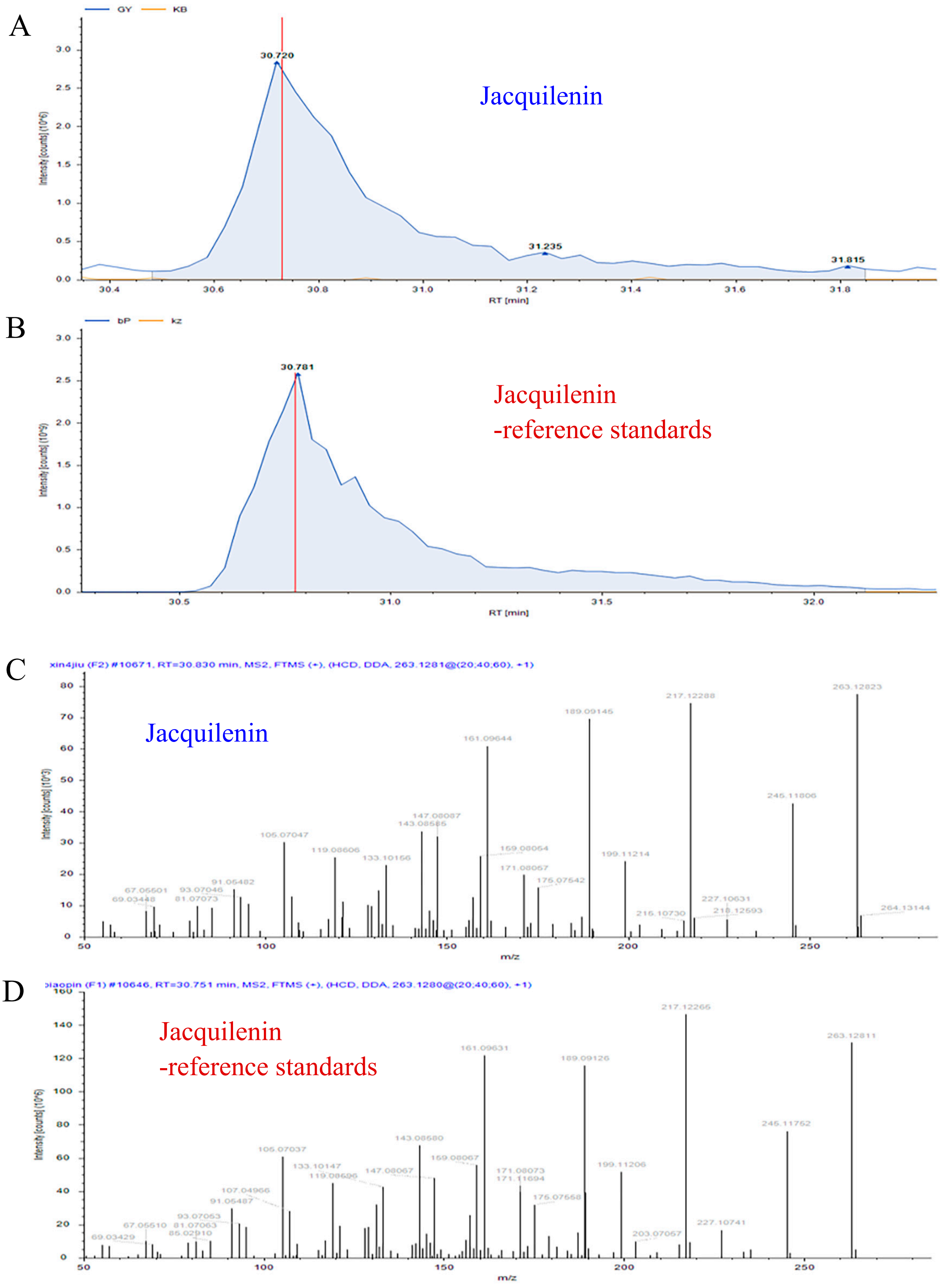

Supplementary Figure S3. (**A**) The ion chromatogram of Jacquilenin detected in positive mode; (**B**) The ion chromatogram of Jacquilenin-reference standards detected in positive mode; (**C**) The secondary mass spectra of Jacquilenin detected in positive mode; (**D**) The secondary mass spectra of Jacquilenin-reference standards detected in positive mode.

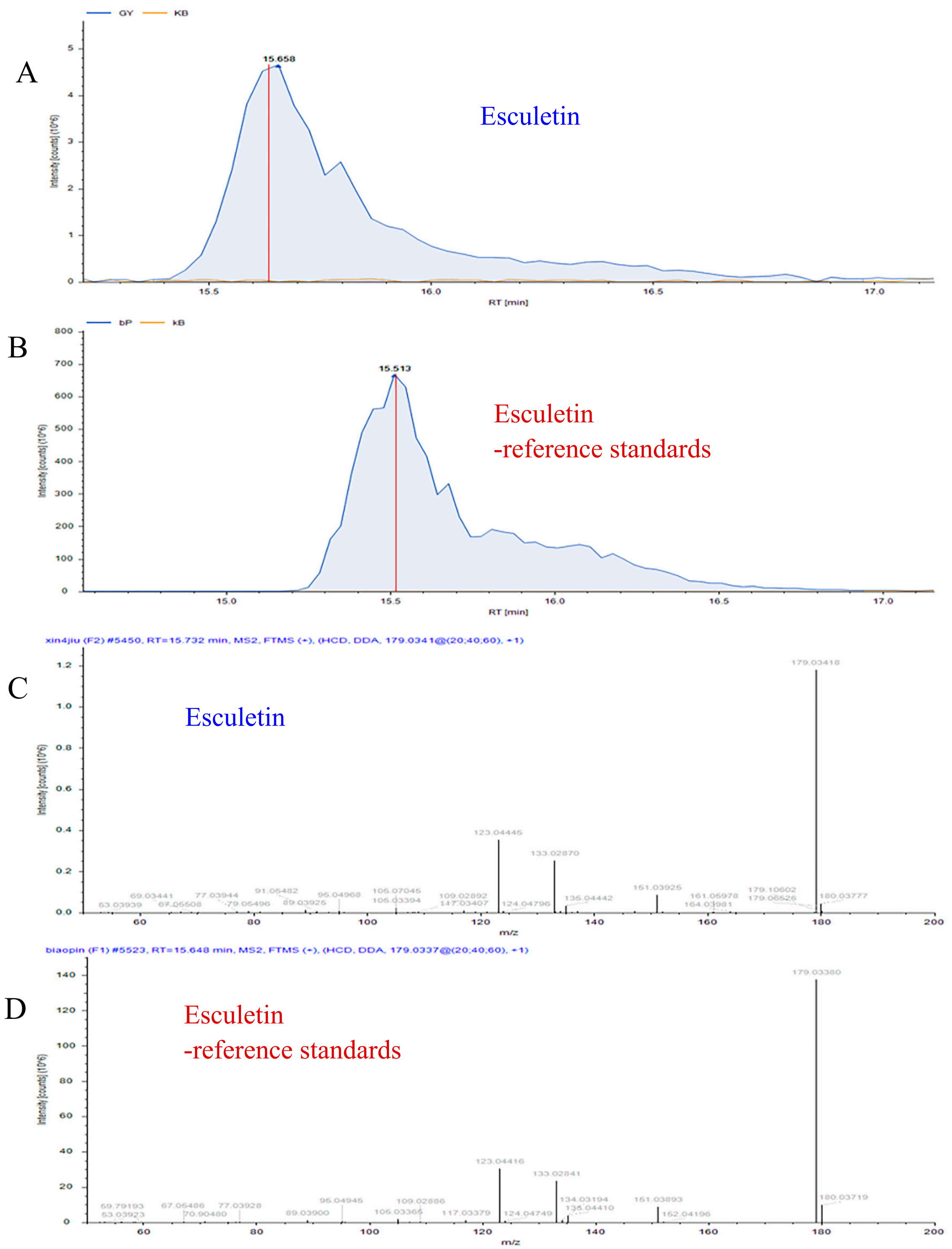

Supplementary Figure S4. (**A**) The ion chromatogram of Esculetin detected in positive mode; (**B**) The ion chromatogram of Esculetin-reference standards detected in positive mode; (**C**) The secondary mass spectra of Esculetin detected in positive mode; (**D**) The secondary mass spectra of Esculetin-reference standards detected in positive mode.

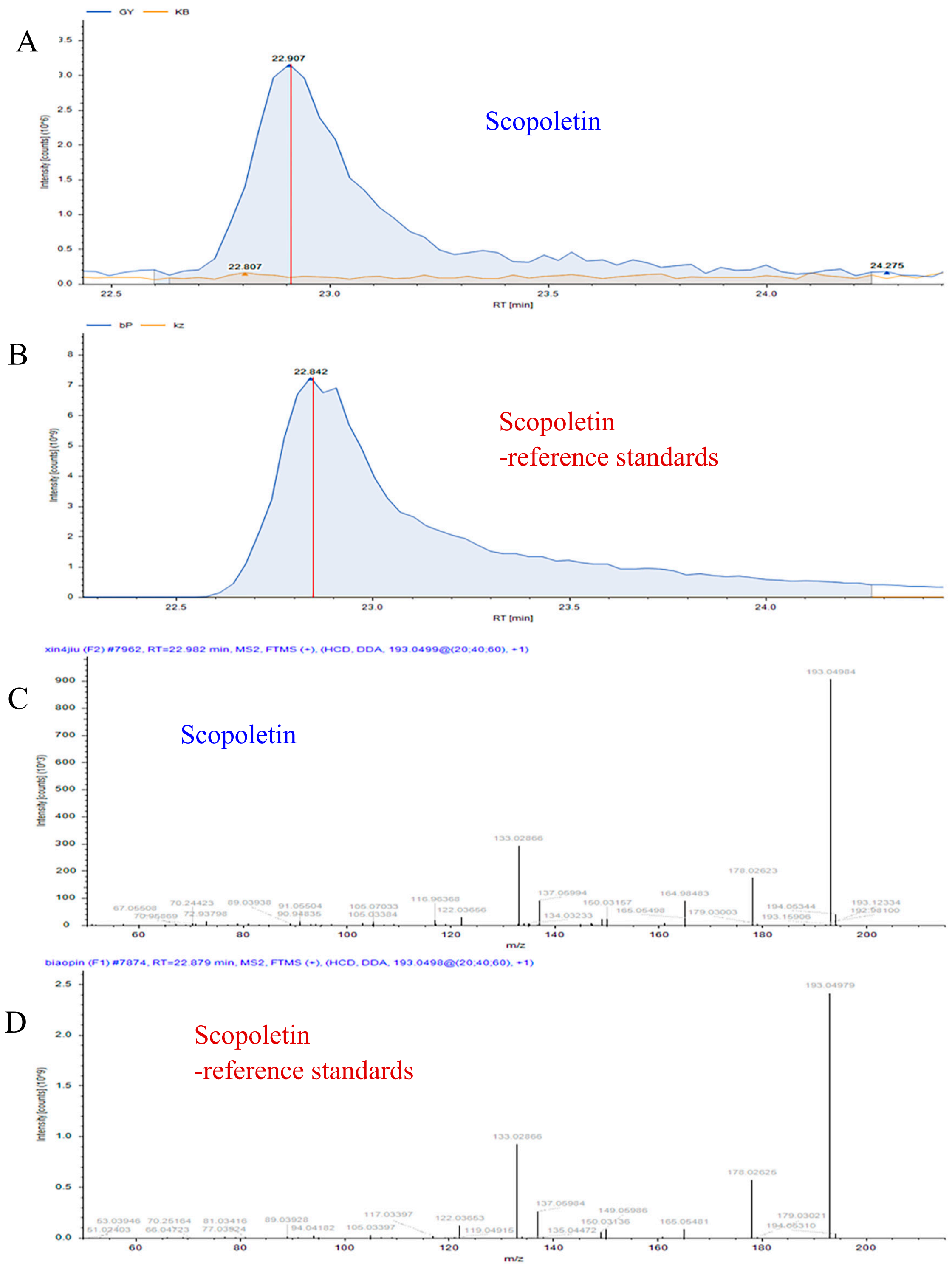

Supplementary Figure S5. (**A**) The ion chromatogram of Scopoletin detected in positive mode; (**B**) The ion chromatogram of Scopoletin-reference standards detected in positive mode; (**C**) The secondary mass spectra of Scopoletin detected in positive mode; (**D**) The secondary mass spectra of Scopoletin-reference standards detected in positive mode.

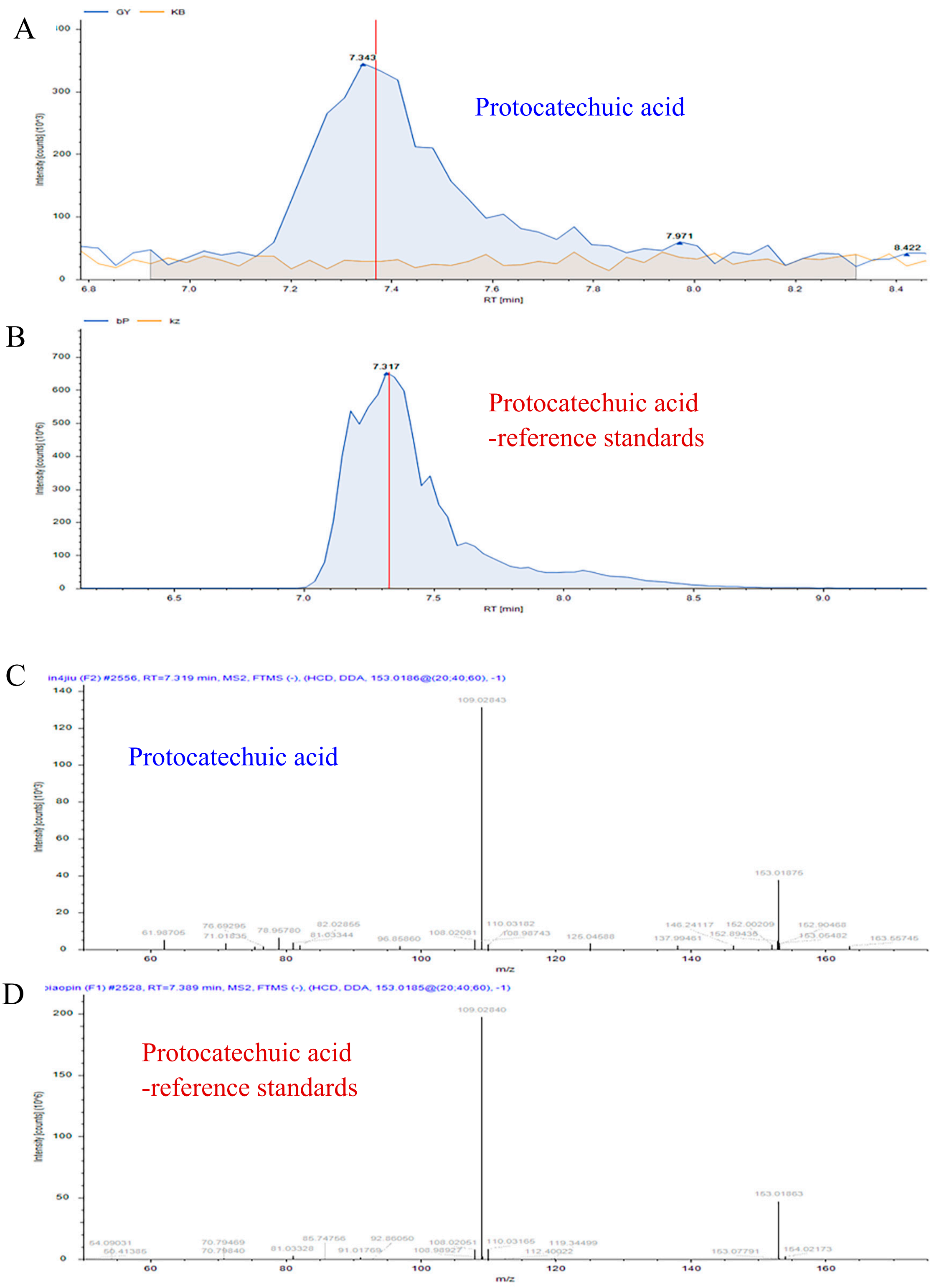

Supplementary Figure S6. (**A**) The ion chromatogram of Protocatechuic acid detected in negative mode; (**B**) The ion chromatogram of Protocatechuic acid-reference standards detected in negative mode; (**C**) The secondary mass spectra of Protocatechuic acid detected in negative mode; (**D**) The secondary mass spectra of Protocatechuic acid-reference standards detected in negative mode.

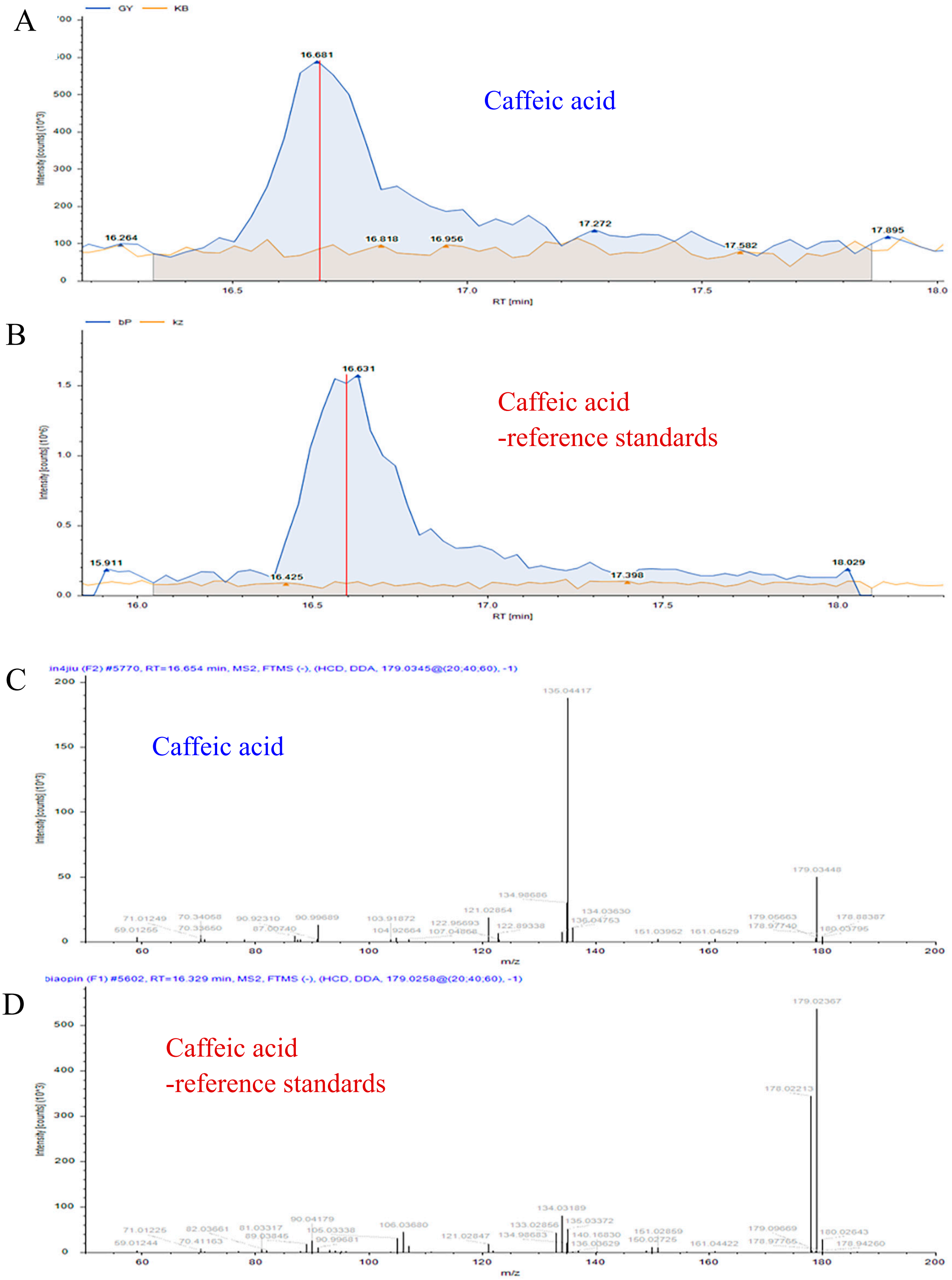

Supplementary Figure S7. (**A**) The ion chromatogram of Caffeic acid detected in negative mode; (**B**) The ion chromatogram of Caffeic acid-reference standards detected in negative mode; (**C**) The secondary mass spectra of Caffeic acid detected in negative mode; (**D**) The secondary mass spectra of Caffeic acid-reference standards detected in negative mode.

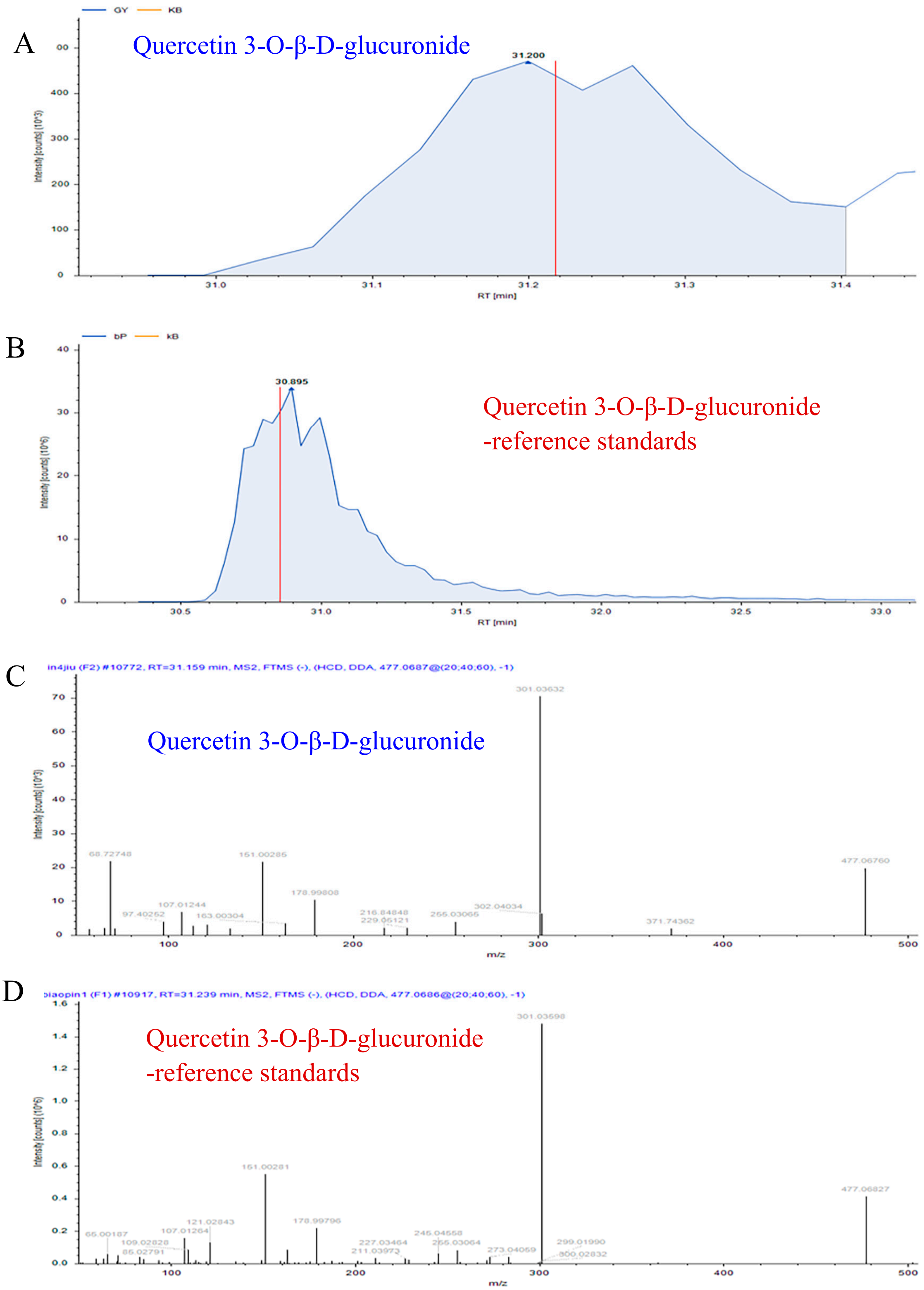

Supplementary Figure S8. (**A**) The ion chromatogram of Quercetin 3-O-β-D-glucuronide detected in negative mode; (**B**) The ion chromatogram of Quercetin 3-O-β-D-glucuronide-reference standards detected in negative mode; (**C**) The secondary mass spectra of Quercetin 3-O-β-D-glucuronide detected in negative mode; (**D**) The secondary mass spectra of Quercetin 3-O-β-D-glucuronide-reference standards detected in negative mode.

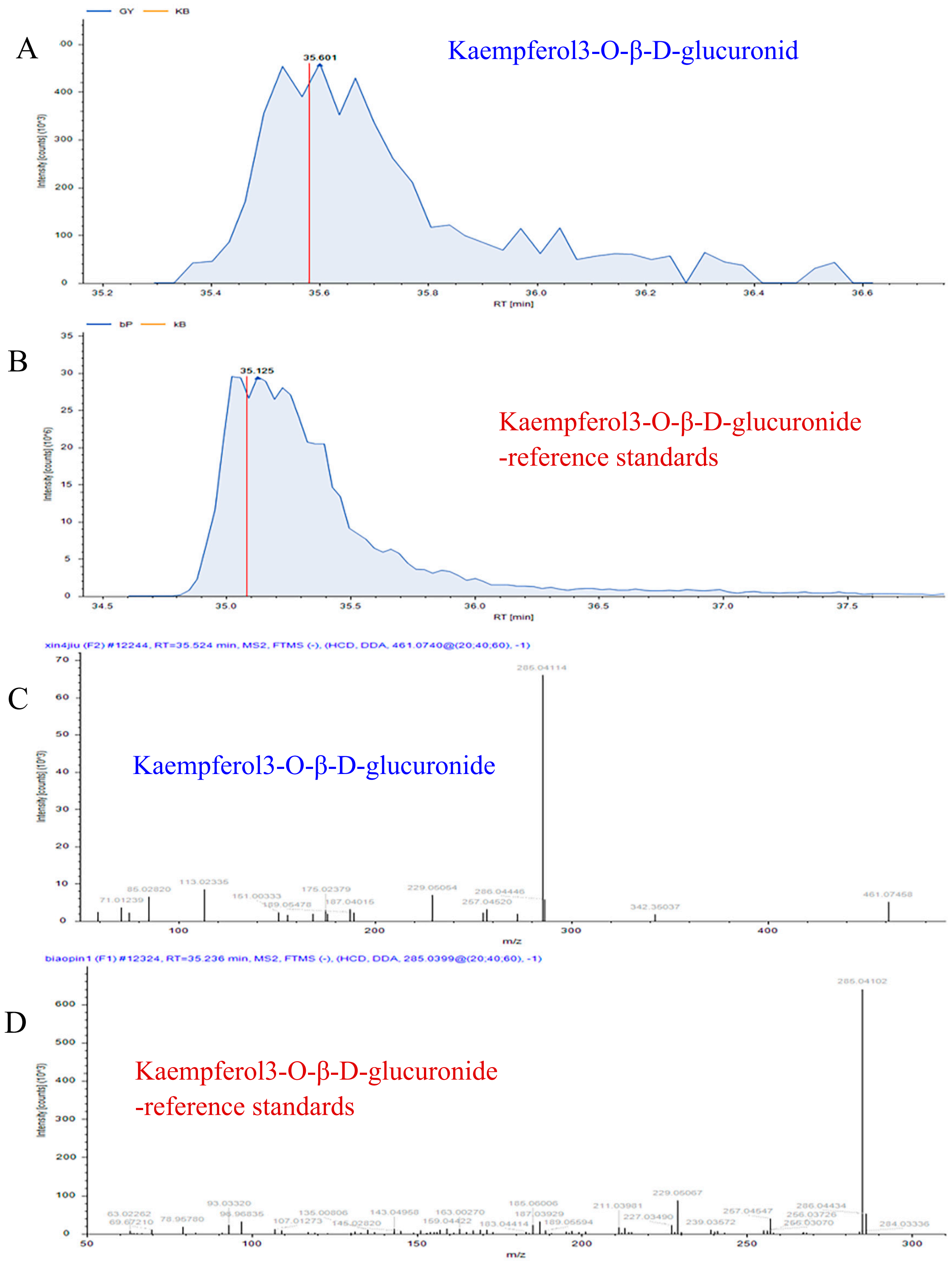

Supplementary Figure S9. (**A**) The ion chromatogram of Kaempferol3-O-β-D-glucuronide detected in negative mode; (**B**) The ion chromatogram of Kaempferol3-O-β-D-glucuronide-reference standards detected in negative mode; (**C**) The secondary mass spectra of Kaempferol3-O-β-D-glucuronide detected in negative mode; (**D**) The secondary mass spectra of Kaempferol3-O-β-D-glucuronide-reference standards detected in negative mode.

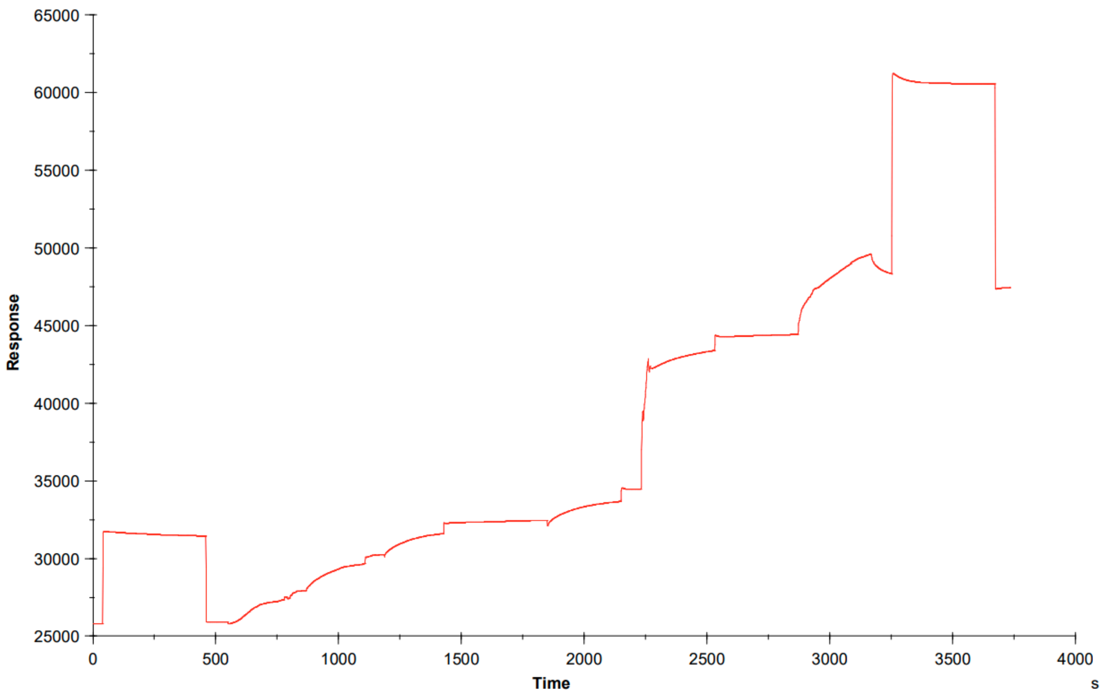

Supplementary Figure S10. UCP2 Protein Coating Diagram.

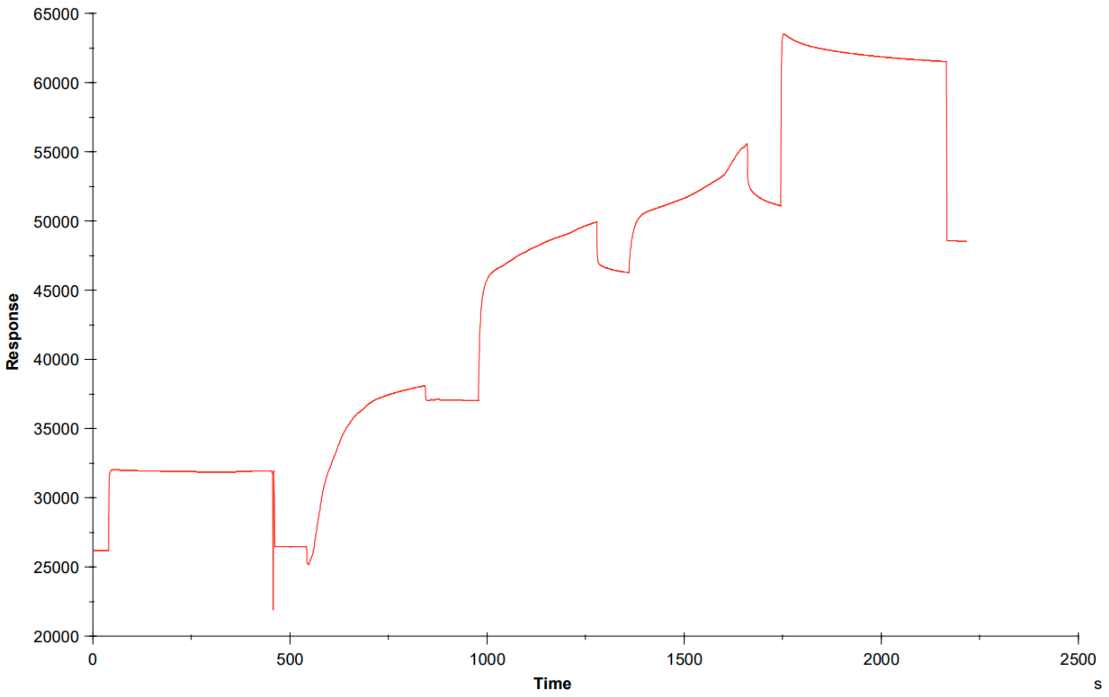

Supplementary Figure S11. NLRP3 Protein Coating Diagram.

Supplementary Table S1. Basic fitting parameters for dimethyl sulfoxide (DMSO) solvent calibration curve.

Included	Cycle	Curve	Chi^2（RU^2）	Y0（RU）
Yes	4	Fc=2-1	0.3797	2.2
Yes	4	Fc=4-3	0.5363	2.9
Yes	35	Fc=2-1	0.6111	5.5
Yes	35	Fc=4-3	0.736	8.1
Yes	45	Fc=2-1	0.4887	7.0
Yes	45	Fc=4-3	0.6483	9.5

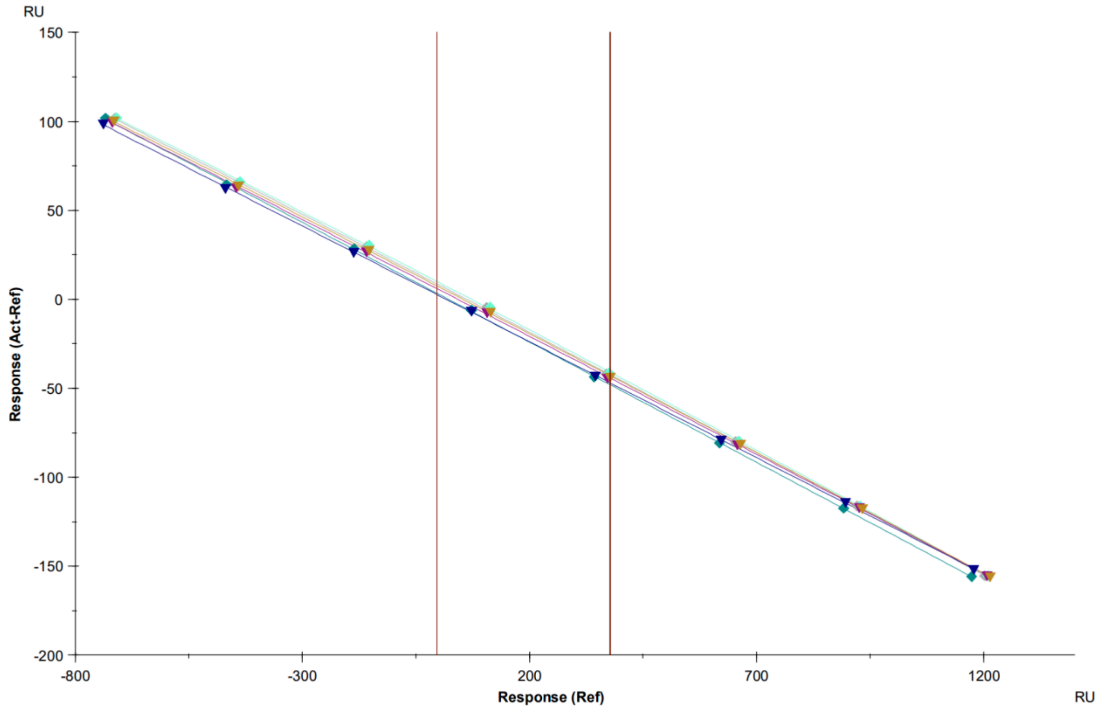

Supplementary Figure S12. DMSO solvent calibration curve chart.
